# Innovations in Microfluidics to Enable Novel Biomedical Applications

**DOI:** 10.3390/bios14100507

**Published:** 2024-10-17

**Authors:** Nan Xiang, Zhonghua Ni

**Affiliations:** School of Mechanical Engineering, Jiangsu Key Laboratory for Design and Manufacture of Micro-Nano Biomedical Instruments, Southeast University, Nanjing 211189, China; nzh2003@seu.edu.cn

As a new technique for precisely controlling micro-/nanoparticles and fluids at the microscale, microfluidics has been attracting increased interest in the fields of material science, medical diagnosis, biological research, and even soft robotics. For use in biomedical applications, microfluidics has been widely employed in areas including biosensing, sample preparation, cell culture, and drug discovery. Some microfluidic products have already been successfully commercialized and have reformed traditional techniques.

Our first Special Issue, “Microfluidics for Biomedical Applications”, was devoted to the most exciting technical innovations in the area of microfluidics, particularly in relation to biomedical applications. We successfully launched the second volume of this Special Issue following the triumph of the first volume. A total of twelve outstanding papers (including seven research articles and five reviews) are included in this Special Issue. We will now briefly introduce these twelve papers.

Cell co-culture is a powerful tool for studying the communications and interactions between cells involved in various cellular activities. Li et al. (contribution 1) review the recent advances in three-dimensional (3D) cell co-culture using microfluidics. They offer new insights into the design of 3D co-culture microfluidic devices and the detection techniques utilized in 3D co-culture microfluidic devices. Finally, they introduce interesting applications for these 3D co-culture microfluidic devices to address real biomedical demands.

The traditional soft lithography technique used for fabricating microfluidic devices is complex and time-consuming and requires a cleanroom environment. With 3D printing, we can rapidly create microstructures or microchannels in bulk materials, and this represents an important alternative for fabricating microfluidic devices. In their review, Li et al. (contribution 2) present a comprehensive summary of the use of light-driven 3D printing techniques to manufacture advanced microfluidic devices. More importantly, they summarize three light-driven 3D printing strategies for creating microfluidic devices for use in cell culture and tissue engineering.

The enrichment of rare circulating tumor cells (CTCs) from peripheral blood is a challenge due to the rarity of CTCs (typically less than 50 CTCs in 1 mL blood) and their large heterogeneity. Sen-Dogan et al. (contribution 3) designed a spiral inertial microfluidic device with hydrofoil-shaped pillars for the high-throughput and label-free separation of CTCs. Their results prove that the new spiral design performs significantly better than the conventional spiral design in terms of the recovery ratio of tumor cells and the depletion ratio of white blood cells. They also analytically validated the device performances using three different tumor cell lines (A549, SKOV-3, and BT-474).

The accurate detection of micro-/nanomaterials (e.g., biomolecules, exosomes, and cells) using resistive pulse sensors represents a promising enabling technology in microfluidics. However, the throughputs of most existing resistive pulse sensors are low. Xu et al. (contribution 4) developed a bipolar pulse-width, multiplexing-based resistive pulse sensor for the high-throughput detection of microparticles. Low errors—2.6% and 6.1%—were achieved for particle sizing and counting, respectively. The practicability of their device was further demonstrated through the detection of HeLa cells.

Diabetes mellitus is an endocrine disease that is common globally and seriously affects human health. Glutamic acid decarboxylase antibody (GADAb) is regarded as a biomarker for the clinical diagnosis of type 1 diabetes. Tao et al. (contribution 5) developed a glass capillary solid-state nanopore for the detection of glutamic acid decarboxylase (GAD65), GADAb, and their antigen–antibody complexes. This glass capillary solid-state nanopore could be employed without any modifications and thus was cost-effective and easy to operate. Based on this nanopore system, the authors successfully achieved the differentiation of GAD65, GADAb, and GADAb-GAD65 complexes.

Magnetic micro-/nanoparticles (MMPs or MNPs) are widely employed in many biomedical applications, as they offer the advantages of high biocompatibility and diverse expansibility. Ger et al. (contribution 6) developed a microfluidic device embedded with a giant magnetoresistance sensor and successfully detected low-concentration MNPs at a velocity of 20 mm/s. A high detection sensitivity of 10 μg/μL for MNPs was achieved using a vertical magnetic field of 100 Oe and a horizontal magnetic field of 2 Oe.

Lunelli et al. (contribution 7) propose a microfluidic scheme using specifically functionalized MMPs inserted in polymeric microchambers. MMPs were functionalized with aptamers, antibodies, or small functional groups for coupling with specific antibiotics. These three different functionalization strategies were carefully compared. The authors found that the functionalization with aptamers allowed them to capture and release almost all tetracycline and to deliver an enriched and simplified antibiotic solution.

Stable and uniform droplet generation at a high throughput is critical in the accurate and efficient detection of digital nucleic acid. Luo et al. (contribution 8) developed a step emulsification microfluidic device with the advantages of flexible droplet generation capability, a small footprint, simple fabrication, low contamination, and high robustness. To increase the uniformity of the generated droplets, a tree-shaped droplet generation nozzle was designed by equating flow rates. Finally, the stable and uniform droplets generated by the step emulsification microfluidic device were employed for nucleic acid amplification and detection.

Intracellular delivery refers to the transportation of substances into cells and is a crucial process in various cellular applications (e.g., drug delivery and gene editing). Among the reported intracellular delivery technologies is mechanoporation, which employs mechanical forces to create temporary pores on cell membranes for delivering substances into cells. Wang et al. (contribution 9) review recent advances in mechanoporation. First, the authors review different mechanoporation technologies and highlight the applications of mechanoporation in stem cell research. They then discuss the integration of mechanoporation into microfluidics for high-throughput intracellular delivery with enhanced transfection efficiency.

Paper microfluidics represents a promising tool for rapid diagnostics and on-site analysis in resource-limited settings, offering the advantages of biodegradability and affordability. Kumar et al. (contribution 10) present a concise overview of paper microfluidics. Sustainable sensing applications of paper microfluidics in healthcare, environmental monitoring, and food safety are explored. Fabrication techniques, principles, and applications in paper microfluidics are also discussed.

The fluidically loaded bi-material cantilever (B-MaC) is a key component of microfluidic paper-based analytical devices. Kumar et al. (contribution 11) studied the dynamics of a B-MaC constructed from Scotch Tape and Whatman Grade 41 filter paper strips. They explored the stress–strain relationship to estimate the modulus of the B-MaC under different saturation levels.

Traditional drug development based on animal experiments is expensive and time-consuming. The organ-on-a-chip (OOC) simulates complex human organ microenvironments and physiological responses in a microfluidic device and offers a promising tool for cost-effective and efficient drug development. Yuan et al. (contribution 12) review the recent advances in OOC systems for drug discovery. Their review focuses on the design, fabrication, sensing capabilities, and applications of OOC systems. Technical challenges in this field are also discussed.

We strongly believe that microfluidics will lead to a revolution in biological research and disease diagnosis in the near future.

